# Mediterranean Diet Favors Vitamin K Intake: A Descriptive Study in a Mediterranean Population

**DOI:** 10.3390/nu16081098

**Published:** 2024-04-09

**Authors:** Ezequiel Pinto, Carla Viegas, Paula Ventura Martins, Catarina Marreiros, Tânia Nascimento, Leon Schurgers, Dina Simes

**Affiliations:** 1 Centro de Estudos e Desenvolvimento em Saúde, Universidade do Algarve, Campus de Gambelas, 8005-139 Faro, Portugal; epinto@ualg.pt (E.P.); tinascimento@ualg.pt (T.N.); 2 Algarve Biomedical Center Research Institute (ABC-RI), Universidade do Algarve, Campus de Gambelas, 8005-139 Faro, Portugal; 3 Centre of Marine Sciences (CCMAR), Universidade do Algarve, Campus de Gambelas, 8005-139 Faro, Portugal; caviegas@ualg.pt (C.V.); cimarreiros@ualg.pt (C.M.); 4 GenoGla Diagnostics, Centre of Marine Sciences (CCMAR), Universidade do Algarve, Campus de Gambelas, 8005-139 Faro, Portugal; 5 Algarve Cyber-Physical Systems Research Centre (CISCA), Universidade do Algarve, Campus de Gambelas, 8005-139 Faro, Portugal; pventura@ualg.pt; 6 Department of Biochemistry, Cardiovascular Research Institute Maastricht, 6200 MD Maastricht, The Netherlands; l.schurgers@maastrichtuniversity.nl

**Keywords:** Mediterranean diet, vitamin K, cross-sectional study, dietary intake

## Abstract

The Mediterranean diet (MD) is associated with improved longevity and the prevention and management of chronic inflammatory diseases (CIDs). Vitamin K, which is present in MD core components such as leafy green vegetables, is also known as a protective factor for CIDs. Estimates of vitamin K intake in Mediterranean settings are still scarce, and the association between MD and vitamin K intake is yet to be established. This study analyzed vitamin K intake and MD adherence in the Algarve region, in Portugal. We conducted a cross-sectional study in a nonrandom sample of adults using an online questionnaire which included a validated food-frequency questionnaire and a screener for MD adherence. A total of 238 participants were recruited (68% women and 32% men). Adherence to the MD was low (11%). Only 10% of the participants had vitamin K intake below the adequate intake. Adherence to the MD was positively correlated with vitamin K intake (r = 0.463; *p* < 0.001) and age (r = 0.223; *p* < 0.001). Our findings underscore the importance of promoting adherence to the MD for optimal vitamin K intake, and future research should focus on developing effective interventions to promote this dietary pattern, particularly among younger individuals and men.

## 1. Introduction

The Mediterranean diet (MD), recognized as an intangible world heritage by UNESCO, is celebrated for its health-promoting qualities and its respect for the environment [1]. While the MD is traditionally associated with countries bordering the Mediterranean Sea, it has been promoted worldwide as one of the healthiest dietary patterns. The MD primarily emphasizes plant-derived foods, including vegetables, fruit, and legumes, while also encouraging the consumption of unrefined cereals, olive oil, fish (over red meat), and moderate amounts of dairy products and red wine [2]. Due to its high nutritional profile, rich in monounsaturated fatty acids (MUFAs), polyunsaturated fatty acids (PUFAs), different antioxidants, and anti-inflammatory compounds, the MD is now acknowledged for its protective effects against chronic inflammatory diseases (CIDs), commonly referred to as age-associated noncommunicable diseases [3]. Numerous prospective observational studies and trials conducted across diverse populations over several decades have consistently demonstrated that adherence to MD offers benefits in preventing all-cause mortality and managing various CIDs. These include cardiovascular diseases (such as coronary heart disease, stroke, and diabetes), bone diseases (including fragility fractures), cancer, neurodegenerative diseases, cognitive health, depression, and respiratory diseases [4].

Interestingly, vitamin K, abundant in core MD components like green leafy vegetables, also plays a protective role in several CIDs, such as cardiovascular disease, bone-related disorders, cancer, dementia, cognitive impairment, and lung diseases [3,5]. The favorable effects of vitamin K may stem from its function as a cofactor for the gamma-carboxylation of vitamin K-dependent proteins (VKDP), which have diverse physiological roles. Additionally, vitamin K exhibits anti-inflammatory, antioxidant, and transcriptional regulatory properties related to osteoblastic genes, cognitive function, antiferroptosis, and tumor progression inhibition [6,7,8].

Vitamin K is a liposoluble vitamin that includes phylloquinone (vitamin K1), mostly found in photosynthetic organisms, including green leafy vegetables, herbs, algae, and vegetable oils, and menaquinones (MKn or vitamin K2), which are primarily produced by bacteria and obtained through the consumption of meat, fermented foods, and dairy products [6,7,8,9]. Vitamin K1 and K2 sources are mainly dietary [10,11], and vitamin K can be recycled through the vitamin K epoxide reductase cycle (VKOR). However, vitamin K2 can also be produced by human microbiota, and MK-4 can be converted from vitamin K at the tissue level. In fact, vitamin K1 accounts for 90% of the total vitamin K in the diet [12], and vitamin K recommended daily intake (RDI) is based on the median intake of vitamin K1. The adequate intake recommendation (adequate intake—AI) for American adults was set to 120 μg/day for men and 90 μg/day for women [13], and in Europe, 75 μg vitamin K has been recommended as the daily allowance (Commission Directive 2008/100/EC).

Mediterranean populations traditionally follow an eating pattern where vitamin-K-rich foods, such as leafy green vegetables and different types of cheeses, are frequently consumed [14,15]; thus, the association between adherence to this dietary pattern and higher vitamin K intake has been mainly assumed but not established [16,17]. Additionally, estimates of vitamin K populational intake within Mediterranean settings are scarce.

It has been reported that adherence to the MD is decreasing, especially among younger inhabitants of Mediterranean regions [18,19,20,21], and recent data for Portugal show that adherence to the MD is low, with prevalences reported at 17% [22] or 8.7% [23]. Given this evidence and recognizing the health benefits of proper vitamin K intake [24,25,26], the aim of the current study was to analyze populational vitamin K intake and MD adherence in the Algarve region, a Mediterranean setting within Portugal. To the best of our knowledge, this is the first study to describe and analyze the association between MD adherence and vitamin K intake in Portugal.

## 2. Materials and Methods

### 2.1. Setting

The Algarve is the southernmost region of Portugal. It spans an area of 4 997 km^2^ and was home to 467,495 residents as of 2021 [27]. It is known for its Mediterranean climate and tourism industry, with many residents employed in tourism-related industries, especially during the peak tourist season, which typically occurs in the summer months. The local economy exhibits diversity, with contributions from sectors such as agriculture, fishing, and small-scale industries [28].

Recent demographic data show that the average age of the population stands at 45.3 years, slightly higher than the national average of 44.9 years. The schooling rate, which gauges the percentage of the population aged 3 to 24 attending educational institutions, was 85.9% in 2020, slightly below the national average of 88.4%. Furthermore, educational attainment among the population aged 15 and over in 2021 shows that only 14% hold a higher education degree [28].

### 2.2. Study Design, Participants, and Data Collection Tools

We conducted a cross-sectional survey in a nonrandom sample of adults using an online, self-fulfillment questionnaire (https://form.jotform.com/222044277445353, accessed on 19 February 2024, NutriK) specifically created for this study, which included questions regarding sociodemographic characteristics, the 14-item Mediterranean Diet Screener, and a previously validated food-frequency questionnaire (FFQ) to assess total vitamin K intake.

The FFQ is composed of 54 food items that contain ≥5 μg of vitamin K/100 g of food or, despite having <5 μg of vitamin K/100 g of food, are commonly included in a Mediterranean diet (soft cheese, boiled chickpeas, pumpkin, bell pepper, mackerel, sardines, almonds, walnuts, and coffee) [29]. Data on the vitamin K content of foods were derived from previous research [12], as well as from both McCance and Widdowson’s and the United States Department of Agriculture’s food composition databases [30,31]. The detailed description of the FFQ and the methods used for its validation are presented elsewhere [32].

Vitamin K intake data provided by the FFQ were presented in μg/day and analyzed considering a 10% threshold around the AI recommendation of 120 μg/day for men and 90 μg/day for women [9]. Thus, an intake of 120 ± 12 μg/day for men and 90 ± 9 μg/day for women was considered as adequate.

The 14-item Mediterranean Diet Adherence Screener (MEDAS) used in this study was the tool created in the “Prevención con Dieta Mediterránea” (PREDIMED) trial [33]. This screener consists of 14 questions related to dietary habits reflecting the key components of the Mediterranean diet, such as the consumption of fruits, vegetables, whole grains, olive oil, nuts, legumes, fish, and moderate alcohol intake, while limiting red meat and processed foods. Each question is scored based on how closely the respondent’s diet aligns with the Mediterranean diet pattern. Scores range between 0 and 14 and provide an indication of adherence to the Mediterranean diet, with higher scores indicating better adherence. Scores ≥10 indicate good adherence to the MD.

Participation in this study was promoted by publicizing the project through social media using the institutional communication channels and tools of the University of Algarve. Inclusion criteria were (i) age ≥ 18 years; (ii) currently living in the region; and (iii) cognitive ability to fulfill the data collection tools. Exclusion criteria were (i) currently under treatment for serious chronic illness, such as cancer or autoimmune diseases; (ii) diagnosis of a disease that impairs nutrition or food choice, such as Crohn’s disease, other malabsorption syndromes, or food allergies.

Sample size calculation, according to methods proposed by Hulley et al. [34] considering a 95% confidence level, a 5% margin of error, and an expected prevalence of adherence to the MD (MEDAS score ≥ 10) of 15%, yielded a minimum sample size of 196 participants. Accounting for a 10% rate for invalid questionnaires, we set the minimum sample size at 216 individuals.

At the end of the data collection phase, 238 valid questionnaires were obtained. Body mass index (BMI) was calculated as the ratio between weight and square of height (kg/m^2^).

All respondents gave informed consent for participation, and the study was conducted according to the guidelines of the Declaration of Helsinki and approved by the ethics committee of the University of Algarve, Faro, Portugal (code CEUAlg Pn°01/2022).

### 2.3. Statistical Analysis

Data were analyzed using IBM SPSS for Windows (version 29.0, 2022, IBM Corporation). Mean, median, standard deviation (SD), and interquartile range (IQR) were computed whenever appropriate, and we used the Kolmogorov–Smirnov test to assess adherence to the normal distribution of quantitative variables (vitamin K intake and age). As the distribution of these variables was non-Gaussian, a nonparametric test (Mann–Whitney) was used for 2-group comparisons. Group comparisons of qualitative variables were assessed with the chi-square test, Fisher’s exact test or, when conditions were not met for their use, with the Fisher–Freeman–Halton exact test. We also computed Spearman’s correlation coefficient for assessing associations between continuous variables.

Statistical significance for all procedures was set at 0.05.

## 3. Results

Of the 238 participants, 68% (*n* = 161) were women and 32% were men (*n* = 77). Men had a higher mean BMI than women and a higher prevalence of excess weight or obesity (Table 1).

Vitamin K intake was higher (*p* = 0.019) in women (Mean = 317.5 ± 23.65 μg/day) than in men (Mean = 222.6 ± 18.45 μg/day). The prevalence of vitamin K intake above AI was also higher (*p* < 0.001) in women (91%; *n* = 146) than in men (74%; *n* = 60).

Adherence to the MD was 11% for all participants: 12% in women and 7% in men. Gender differences in total adherence to MD were statistically not significant (*p* = 0.284), but the MEDAS score was higher in women (*p* = 0.009). This can be explained by gender differences in some specific MD habits: comparatively with women, men report lower use of olive oil for cooking (86% vs. 97%; *p* = 0.001), a higher prevalence of red/processed meat intake at least once a day (74% vs. 58%; *p* = 0.001), a higher prevalence of soda drink intake at least once a day (35% vs. 19%; *p* = 0.005), and a higher prevalence of at least seven glasses of wine a week (14% vs. 5%; *p* = 0.013).

In our population, participants with MD adherence were significantly different from nonadherents in most dietary habits reflecting the key components of the MD, except for the use of olive oil as the main lipid (*p* = 0.147), the infrequent intake of commercial sweets and confectionery (*p* = 0.097), the more frequent poultry intake than red meats (*p* = 0.166), and the frequent use of sofrito sauce (*p* = 0.520) (Table 2).

Participants who adhered to the MD had a significantly higher intake of vitamin K (M = 528.5 ± 389.8 μg/day) than nonadherents (M = 257.2 ± 232.1 μg/day) (Table 3).

Additionally, there is a statistically significant positive correlation between vitamin K intake and both the MEDAS score (r = 0.463; *p* < 0.001) and age (r = 0.223; *p* < 0.001). The association between vitamin K intake and MEDAS score is maintained when controlling for age (r = 0.352; *p* < 0.001), both in men (r = 0.455; *p* < 0.001) and in women (r = 0.315; *p* < 0.001), suggesting the significant role of MD habits in vitamin K intake, independent of age and gender.

Vitamin K intake did not correlate with BMI (r = 0.003; *p* = 0.958).

The food items which contributed most to vitamin K intake are presented in Table 4. We did not find any gender differences in specific food items contributing to total vitamin K intake (*p* > 0.05).

## 4. Discussion

In this work, for the first time, we report a correlation between adherence to MD and vitamin K intake in a Portuguese population residing in the Algarve region. Among the participants, 85% exhibited a vitamin K intake of at least 10% above the RI. Specifically, the mean daily vitamin K intake was 317.5 ± 23.65 μg/day for women and 222.6 ± 18.45 μg/day for men.

Globally, estimates of vitamin K intake reveal significant variations across countries and divergent findings among studies. These disparities can be explained by factors such as different methodological approaches for estimations and dietetic patterns [35,36,37,38,39,40]. A comprehensive assessment of vitamin K intake in Europe, performed by the EFSA Panel on Dietetic Products, Nutrition, and Allergies (NDA), estimated a mean total vitamin K intake ranging between 72 and 196 ug/day in adults aged 18 and above, drawing from data available in Finland, France, Ireland, Italy, Netherlands, Sweden, and the UK [41]. Interestingly, Italy and the Netherlands reported higher mean intake estimates, surpassing 150 μg/day [41]. In the Netherlands, where a high intake of vegetables is part of the dietetic pattern, vitamin K intake was the highest.

Existing studies predominantly focus on vitamin K1 and menaquinones intake, which range between 230–288 ug/day for vitamin K1 and 27–31 ug/day for menaquinones [26,42,43,44]. Our analysis was limited to total vitamin K due to the scarcity of nutritional composition data and the absence of a validated tool to quantify both vitamin K1 and vitamin K2 intake through self-reported data. Nevertheless, our overall vitamin K estimates align with those observed in the Netherlands. Although vitamin K1 has been demonstrated as the major source of nutritional vitamin K [43], future research should be conducted to distinguish between K1 and K2 dietary sources, physiological functions, and contribution to an MD.

While there are clear overlapping patterns between MD and food items with high vitamin K content, particularly K1 in green vegetables and in olive oil, studies to assess the association between MD and vitamin K intake are, to the best of our knowledge, unknown. One study performed in Italy, comparing vitamin K1 levels between hemodialysis patients and controls, reported a mean of 129.2 ug/day (61.5–380.5) for vitamin K1 in the control group [45]. Although Italy is a Mediterranean country, the authors did not specifically explore the connection between MD adherence and vitamin K1 intake. Additionally, research on the influence of MD on anticoagulation therapy involving vitamin K antagonists has been conducted; however, a direct association between vitamin K intake and adherence to an MD was not established in these studies [16,17]. This relation would potentially add an increment of clinical value to the MD, since vitamin K deficiency has been widely reported to be associated with vascular [46] and renal disease [47].

The question of whether established vitamin K RI is adequate to fulfill vitamin K requirements is still a matter of debate in the scientific community. The RI was based on the maintenance of normal hemostasis, with sufficient vitamin K to maintain proper gamma-carboxylation of blood coagulation factors. However, several extrahepatic VKDP proteins have been shown as incompletely gamma-carboxylated in healthy adults not taking vitamin K supplements [48], with implications in several CIDs [reviewed in [6,7]]. In fact, it was suggested that the Western diet does not contain sufficient vitamin K to fulfill the gamma-carboxylation of extrahepatic tissues, such as the vascular system and bones [48]. Although several interventional clinical trials have established the beneficial effects of vitamin K supplementation in bone and vascular health, others have reported a lack of effects [49,50,51,52,53,54,55,56]. This can be explained by the lack of standardized protocols, the use of different vitamin K vitamers, different follow-up and endpoint measurements, and different approaches to determining vitamin K levels [6,7]. Additional long-term vitamin K interventional studies in healthy subjects and disease cohorts are required to fully understand the optimal vitamin K levels and the impact of vitamin K supplementation on health status.

In our study, we observed a significant association between lower vitamin K intake and poorer adherence to the Mediterranean diet (MD). This finding leads us to hypothesize that promoting the MD could potentially enhance vitamin K intake. However, it should be noted that despite low adherence to the MD, some participants achieved adequate or above-average vitamin K intake, particularly among women. This phenomenon might be partially explained by research indicating that dietary patterns with reduced meat consumption can still facilitate the intake of vitamin-K-rich foods, even if they deviate from the traditional MD [24,46]. Nevertheless, it is crucial to note that these alternative diets may lack the comprehensive health benefits associated with adherence to the MD.

A large body of evidence associates diverse health benefits with higher adherence to the MD, including decreased mortality and prevention of cardiovascular diseases, diabetes, cancer, and neurogenerative diseases [57]. Furthermore, the MD principles align with healthier lifestyles, potentially influencing other healthy choices and, nutritionally, promoting a synergistic interplay, as the MD is not just a collection of isolated nutrients [57,58,59].

Olive oil, one of the main features of MD, might enhance vitamin K absorption, while fiber provided by adequate amounts of vegetables could slow down digestion, allowing for better utilization [60]. This combined effect could amplify the benefits of individual components, such as vitamin K.

Adherence to the MD for all participants was 11%, lower than the one estimated by the last National Food, Nutrition, and Physical Activity Survey of the Portuguese General Population [61], showing adherences of 18% (95% CI: 17.3–19.1) at the national level and 20% for Algarve. The data for this national survey were collected in 2015–2016, and recent studies suggest that MD adherence has been declining, with estimates that only around 10–15% of adults maintain most dietary habits associated with an MD. The results of the same study, reporting adherence to the MD and its association with country-specific socioeconomic characteristics, suggest that the level of adherence may be influenced by socioeconomic factors and, thus, decreased during the recent economic crisis [20,62].

Our results on MD adherence are also aligned with the literature, suggesting that adherence to the MD is lower in men and in younger individuals [19,63]. In our study, men had a significantly lower (*p* = 0.009) MEDAS score (M = 6.3 ± 2.16) than women (7.1 ± 2.15), but it is important to note that these results could be biased due to the low representativeness of men in our sample. In addition, the positive correlation between MEDAS score and age suggests that older participants have a higher adherence to the MD.

The literature also suggests that the dietary features of the MD that are most commonly being lost seem to be mainly driven by excess meat consumption [64]. Our data support these claims, with low adherence to MD coexisting with a high intake of red/processed meats (63% of participants reported an intake of at least once a day).

We obtained 238 valid replies to our questionnaire, mostly from women (68%). This number of participants surpassed the minimum estimated to achieve a representative sample, according to our calculations, but some sociodemographic characteristics of our participants differ from the available populational data for the Algarve region.

The gender distribution for the participants was 68% for women and 32% for men. Census data for Portugal [65] show that the gender distribution in the Algarve, for adults, is approximately 52% women and 48% men.

Our participants also had a significantly higher rate of higher education than the estimated for the Algarve region: 65% of all participants (70% in women and 55% in men) had concluded some higher education degree, contrasting with the data from the 2021 census showing a rate of 44% for Portugal and just 8% for the Algarve, which is well below the national average. This may have introduced some bias in our results and is in accordance with some research showing that women, especially those with a higher educational level, are generally more likely to participate in health and nutrition studies compared with men [66,67]. It is suggested that women tend to be more interested in health topics, more actively seek health information, and may perceive themselves at higher risk for certain health conditions and thus be more motivated to participate in research related to those conditions [66]. Women may also be subjected to cultural beliefs, traditions, norms, and values that increase their feeling of being helpful and contributing to health knowledge [68,69].

Our participants reported heights and weights that resulted in a 33% combined prevalence for preobesity (BMI between 25–29.9 kg/m^2^) and obesity (BMI ≥ 30 kg/m^2^), significantly higher (*p* = 0.0037) in men (43%) than in women (29%). These data are below national and regional estimates from the last populational survey [61]. Data available from adults at the national level suggest rates of 36.5% of preobesity and 21.6% for obesity, i.e., around 58% for the combined prevalence of preobesity and obesity. The same survey suggests, in Algarve, rates of 36.3% for preobesity and 19.2% for obesity, resulting in around 56% combined prevalence. The underestimation of weight is a well-documented phenomenon in the scientific literature across various populations and settings, which makes a case for the limited use of self-reported anthropometric measurements in health research [70,71]. However, due to the methodology applied in our study, this was considered the only way to have a general characterization of our participants. We believe that our results are not significantly biased, as it was not an objective of our research to perform a proper assessment of nutritional status.

Despite the limitations of our research related to the cross-sectional design that precludes analyzing causality, the self-reported intake assessment, and the nonrandom nature of our sample, we found a significant association between adherence to MD and vitamin K intake. Additional research, employing larger and more diverse samples, as well as longitudinal designs with biomarkers and dietary records, could enhance the validity of these findings and provide a deeper understanding of the relationship between dietary patterns, vitamin K intake, and health outcomes.

## 5. Conclusions

Our results indicate that adherence to the MD is low, particularly among younger individuals. Vitamin K intake for most participants is either comparable to or exceeds the recommended adequate intake. Men exhibit both lower adherence to the MD and a higher prevalence of values below the recommended intake.

Our data suggest a positive association between MD adherence and vitamin K intake, indicating that the MD favors intake of vitamin K. Furthermore, our findings underscore the significance of promoting adherence to the MD, especially among individuals with low vitamin K intake.

Future research should prioritize developing effective interventions to promote this dietary pattern, particularly among younger individuals and men, to mitigate the risk of age-related diseases.

Considering the scientific evidence linking improved adherence to MD with health benefits and environmental sustainability, future research and initiatives should raise public awareness about the health and environmental advantages associated with higher MD adherence. Additionally, efforts should focus on enhancing accessibility to the MD for diverse communities worldwide.

## Figures and Tables

**Table 1:** Sociodemographic characteristics, body mass index (BMI), vitamin K intake, and adherence to the Mediterranean diet (MD).

Variable	All (*n* = 238)	Women (*n* = 161)	Men (*n* = 77)	*p*-Value
Age (years); M ± SD	37.5 ± 1.01	37.2 ± 1.16	38.0 ± 1.99	0.984 *
BMI (kg/m^2^); M ± SD	23.8 ± 0.25	23.4 ± 0.31	25.0 ± 0.43	**0.003** *
Preobese or obese (BMI ≥ 25); %; *n*	33%; 80	29%; 47	43%; 33	**0.037** **
Education:				
Less than high school level; %; *n*	1%; 2	-	3%; 2	**0.013** ***
High school; %; *n*	35%; 82	30%; 49	43%; 33	
Higher education; %; *n*	65%; 154	70%; 112	55%; 42	
Vitamin K intake (μg/day); M ± SD	285.2 ± 17.05	317.51 ± 23.65	222.57 ± 18.45	**0.019** *
Vitamin K adequate intake:				
Below; %; *n*	10%; 24	4%; 6	22%; 28	**<0.001** **
±10%; %; *n*	5%; 12	6%; 9	4%; 3	
Above; %; *n*	85%;206	91%;146	74%; 60	
Adherence to MD; %; *n*	11%; 26	12%; 20	7%; 6	0.284
MEDAS Score:				
Range (Min–Max)	1- 12	2- 12	1- 12	
M ± SD	6.8 ± 2.18	7.1 ± 2.15	6.3 ± 2.16	
Md; IQR	7; 3	7; 3	6; 4	**0.009** *

M—mean; SD—standard deviation; Md—Median; IQR—interquartile range; * Mann–Whitney’s test; ** chi-square test; *** Fisher–Freeman–Halton’s test; statistical significance (*p* < 0.05) is highlighted in **bold**.

**Table 2:** Dietary habits according to adherence to the Mediterranean diet (MD).

Mediterranean Dietary Habits	All (%) (*n* = 238)	Adherence to MD (%) (*n* = 26)	Nonadherence to MD (%) (*n* = 212)	*p*-Value
Use of olive oil as main culinary lipid	93	100	92	0.147
Olive oil >4 tablespoons	21	50	17	**<0.001**
Vegetables ≥2 servings/day	52	92	47	**<0.001**
Fruits ≥3 servings/day	28	58	25	**<0.001**
Red/processed meats <1/day	37	88	30	**<0.001**
Butter, cream, margarine <1/day	45	73	41	**0.002**
Soda drinks <1/day	76	100	73	**0.002**
Wine glasses ≥7/week	8	23	6	**0.003**
Legumes ≥3/week	34	77	29	**<0.001**
Fish/seafood ≥3/week	37	65	33	**0.001**
Commercial sweets and confectionery ≤2/week	75	88	74	0.097
Tree nuts ≥3/week	27	69	22	**<0.001**
Poultry more than red meats	69	81	67	0.166
Use of sofrito sauce ≥2/week	80	85	79	0.520

*p*-value for comparisons between adherent and nonadherent groups computed with the chi-square test; statistical significance (*p* < 0.05) is highlighted in **bold**.

**Table 3:** Vitamin K intake according to adherence to the Mediterranean diet (MD).

Vitamin K intake	Adherence to MD (%) (*n* = 26)	Nonadherence to MD (%) (*n* = 212)	*p*-Value
Intake (μg/day); M ± SD	528.5 ± 389.8	257.2 ± 232.1	**0.002** *
Adequate intake:			
Below; %; *n*	-	11%; 23	0.096 **
±10%; %; *n*	-	5%; 11	
Above; %; *n*	100%; 26	84%; 178	

M-mean; SD—standard deviation; * Mann–Whitney’s test; ** Fisher–Freeman–Halton’s test; statistical significance (*p* < 0.05) is highlighted in **bold**.

**Table 4:** Foods that contributed the most to total vitamin K intake, presented as mean percentage of total vitamin K intake, in all participants and by gender.

Food	Contribution (%) to Total Vitamin K Intake M ± SD			
	All (*n* = 238)	Women (*n* = 161)	Men (*n* = 77)	*p*-Value *
Spinach	20 ± 15	22 ± 16	17 ± 12	0.092
Turnip greens, creamed spinach, green vegetable puree	19 ± 14	19 ± 15	19 ± 13	0.896
Broccoli	14 ± 12	13 ± 11	15 ± 13	0.037
Lettuce or mixed green salad	7 ± 8	7 ± 8	7 ± 7	0.217
Cabbage, collard greens, Brussels sprouts	4 ± 5	4 ± 5	3 ± 4	0.754
Stir-fry or stew with vegetables and/or pasta	3 ± 4	3 ± 5	2 ± 2	0.538
Coriander or parsley	3 ± 3	2 ± 3	3 ± 4	0.083
Olive oil	2 ± 5	2 ± 4	3 ± 6	0.323
Watercress and arugula	2 ± 3	2 ± 3	2 ± 3	0.291

M—mean; SD—standard deviation; * Mann–Whitney’s test for gender differences.

## Data Availability

The data presented in this study are available on request from the corresponding author due to privacy restrictions.
